# AI‐Based Unmixing of Medium and Source Signatures From Seismograms: Ground Freezing Patterns

**DOI:** 10.1029/2022GL098854

**Published:** 2022-08-11

**Authors:** René Steinmann, Léonard Seydoux, Michel Campillo

**Affiliations:** ^1^ ISTerre Équipe Ondes et Structures Université Grenoble‐Alpes UMR CNRS 5375 Gières France; ^2^ Department of Earth, Atmospheric and Planetary Sciences Massachusetts Institute of Technology Cambridge MA USA

**Keywords:** seismology, artificial intellegence, blind source separation, unsupervised learning, time series analysis

## Abstract

Seismograms always result from mixing many sources and medium changes that are complex to disentangle, witnessing many physical phenomena within the Earth. With artificial intelligence (AI), we isolate the signature of surface freezing and thawing in continuous seismograms recorded in a noisy urban environment. We perform a hierarchical clustering of the seismograms and identify a pattern that correlates with ground frost periods. We further investigate the fingerprint of this pattern and use it to track the continuous medium change with high accuracy and resolution in time. Our method isolates the effect of the ground frost and describes how it affects the horizontal wavefield. Our findings show how AI‐based strategies can help to identify and understand hidden patterns within seismic data caused either by medium or source changes.

## Key Points

- With methods of unsupervised learning, we identify source and medium processes in seismograms
- A data‐driven product of the seismogram tracks a continuous medium change due to freezing and thawing of the surface
- The data‐driven product can act as a filter and reveal the hidden signature of the medium change

## 1 Introduction

Continuous seismograms are time series of the ground motion recorded at a single location and provide a vast amount of information about processes occurring at the Earth's surface and interior. The recorded ground motion at a given location results from the convolution of the medium's impulse response—expressed as the Green's function—and the seismic waves emitted by various sources, often simultaneously. Thus, continuous seismograms are goldmines to study the medium's properties or sources in time. However, unmixing source or medium changes is often not easy, especially if source and medium changes coincide. For instance, seismic recordings in the vicinity of volcanoes, where many different source and medium effects occur, are challenging and complex datasets to analyze.

To better explore continuous seismic data, seismologists developed many data processing tools to extract valuable information for the task at hand. For example, the short‐term‐average to long‐term‐average energy ratio (STA/LTA) scans the continuous recordings for impulsive signals (Allen, 1978). On the other hand, passive image interferometry can interrogate the medium regularly by exploiting the ambient seismic signals of a data set (Sens‐Schönfelder & Wegler, 2006). Undoubtedly, these tools delivered many new insights into the processes happening at and inside the Earth. However, it is important to note that the design of the tools and the related preprocessing favors certain processes in the seismic data. This can be a problem if the source or medium processes encoded in the seismic data are poorly understood. For example, non‐volcanic tremors were detected about 20 years ago (Obara, 2002), and still today, the physical mechanism and signal properties of such events are not well apprehended. Therefore, it remains unclear if these signals do not exist in specific environments or if the detection tools are not adapted to the task (Bocchini et al., 2021; Pfohl et al., 2015).

Artificial intelligence (AI) can help overcome those blind spots and discover new signals or hidden patterns within the data. Recently, clustering gained attention as a method to identify families of signals in the continuous seismograms (Holtzman et al., 2018; Jenkins et al., 2021; C. W. Johnson et al., 2020; Köhler et al., 2010; Mousavi et al., 2019; Seydoux et al., 2020; Snover et al., 2020; Steinmann et al., 2022). In the most common approach, characteristics—often called features—are calculated for a sliding window. Then, clustering algorithms perform a similarity measurement within the set of characteristics and assign a cluster to each window. Until now, the applications showed that this approach mainly identifies families of signals related to source processes such as geothermal activity (Holtzman et al., 2018), different types of anthropogenic activity (Snover et al., 2020), seismic background activity (C. W. Johnson et al., 2020) or precursory signals of a landslide (Seydoux et al., 2020). To our knowledge, medium changes have been disregarded so far in this task.

In the present study, we make the first attempts toward inferring not only source processes but also medium changes from continuous single station seismograms in a data‐driven fashion.

## 2 A Thin Ground Frost Layer Visible in Temperature Data and Seismic Velocity Variations

**Figure 1:**
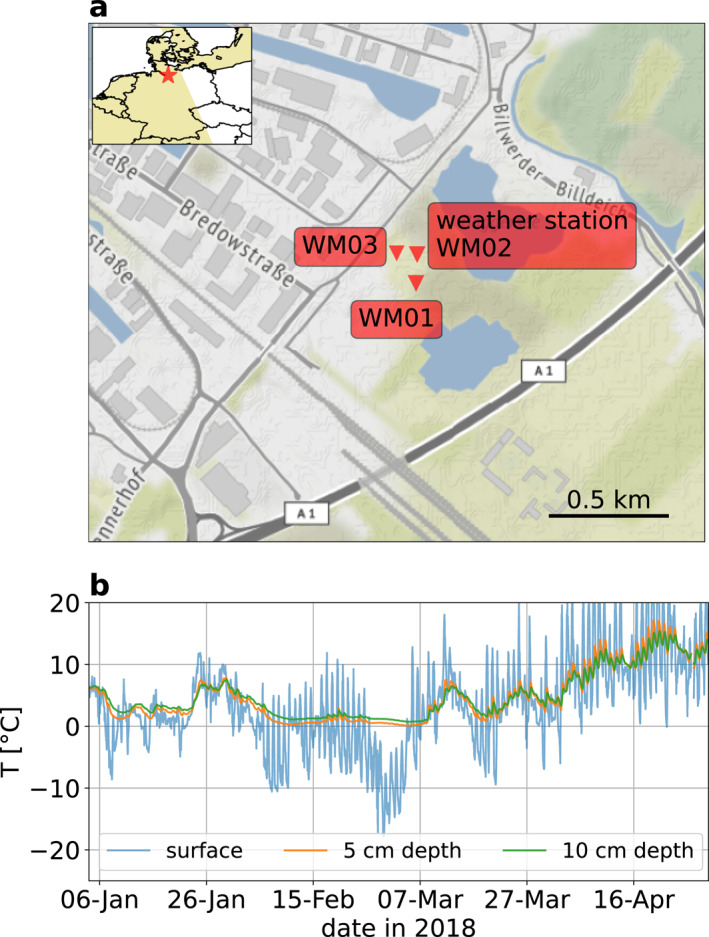
Temperature and seismic stations used in the study. (a) Map of the measuring site in Hamburg, Germany, with the three broadband and three‐component seismic sensors WM01, WM02, and WM03. (b) Temperature time series measured at the surface, 5 and 10 cm depth close to station WM02 with a sampling period of 10 min.

The study site is located in the city of Hamburg, Germany (Figure 1a). Besides the three broadband sensors WM01, WM02, and WM03, the site includes various meteorological sensors near station WM02. At 5, 10 , 80 , and 120 cm depth and at the surface, temperature sensors deliver a measurement every 10 min Figure 1b depicts the temperature time series at the surface, 5  and 10 cm depth from 4 January 2018 to 30 April 2018. Until the end of March, the air temperature ranges between −20°C and 20°C indicating a continuous freezing and thawing of the near‐surface. In particular, the end of February is a cold period with freezing air temperature during daytime and nighttime. However, at 5 and 10 cm depth, the sensors do not reach below 0°C and do not follow the air temperature as they do later in March. This is known as the zero‐curtain effect: the phase change from water to ice in the soil releases latent heat, which causes the freezing process to slow down (Outcalt et al., 1990). This implies that the ground frost is not deeper than 5 cm during the coldest period.

The freezing and thawing process on a centimeter scale was well tracked with seismic velocity variations retrieved from passive image interferometry applied to the data from the three broadband stations WM01, WM02, and WM03 (Steinmann et al., 2021). Freezing periods caused a velocity increase and thawing periods caused a velocity decrease. The local seismic wavefield comprises many non‐stationary seismic sources related to the anthropogenic activity, such as commuter and freight trains in the south, a highway passing in the southeast (labeled A1 on Figure 1a), a close gravel pit (marked by the two nearby lakes on Figure 1a) and an industrial neighborhood in the northwest. The combination of the continuously changing medium due to the freezing and thawing and many non‐stationary seismic sources makes it an interesting study case for our approach to disentangle the medium from the source effects blindly.

## 3 Seismic Pattern Detection With Hierarchical Waveform Clustering

**Figure 2:**
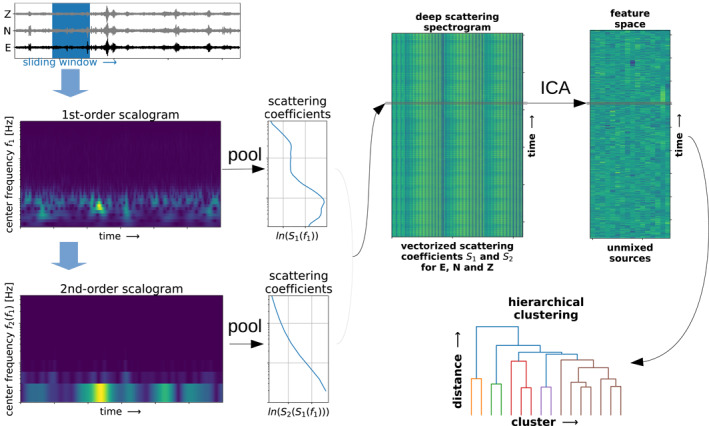
Sketch of the hierarchical waveform clustering approach. A two‐layer scattering network with wavelet transforms, modulus and pooling operations calculates the deep scattering spectrogram. An independent component analysis extracts the most relevant features, which are used for hierarchical clustering.

We search for the imprint of the ground frost within the continuous three‐component seismograms recorded by a single station with the hierarchical waveform clustering approach introduced in Steinmann et al. (2022). Hierarchical clustering observes how a data set merges into clusters based on some similarity criterion (Estivill‐Castro, 2002). In our case, we calculate the similarity between waveforms from a set of features derived from a deep scattering spectrogram, as depicted in Figure 2. First, we calculate the deep scattering spectrogram of the continuous three‐component seismograms with a deep scattering network, as introduced in Andén and Mallat (2014) and adapted to seismology in Seydoux et al. (2020). A deep scattering network is a deep convolutional neural network, where the convolutional filters are restricted to wavelets and the activations to modulus operation. We choose Gabor wavelets as originally proposed in Andén and Mallat (2014) and do not learn the wavelets as the authors did in Seydoux et al. (2020). The output of such a network at each layer allows building the deep scattering spectrogram representation of a continuous multichannel seismogram. This representation of time series is relevant for classification purposes since it preserves signal phenomena such as attack and amplitude modulation. Moreover, a deep scattering spectrogram is locally translation invariant and stable toward small‐amplitude time warping deformations (Andén & Mallat, 2014). Indeed, Steinmann et al. (2022) showed that hierarchical waveform clustering performs poorer if the deep scattering spectrogram is replaced by a Fourier‐based spectrogram. We depict a two‐layer scattering network in Figure 2, where we apply a sliding window on a single‐component seismogram and calculate the first‐order scalogram with the wavelet transform. A second wavelet transform is applied to the first‐order scalogram creating the second‐order scalogram. A pooling operation collapses the time axis of the scalograms and recovers the first‐ and second‐order scattering coefficients. For each component of the ground motion record, we calculate the scattering coefficients and concatenate them. We repeat this for each window and retrieve the deep scattering spectrogram. The design of the scattering network (number of wavelets, type of pooling, etc.) can be adapted to the task at hand and is explained more in detail in Text S1 in Supporting Information S1.

Deep scattering spectrograms are redundant and high‐dimensional representations, not directly suited for clustering due to the curse of dimensionality (Bellman, 1966). Therefore, we extract the most relevant characteristics—or features—and reduce the number of dimensions with an independent component analysis (ICA), a linear operator for feature extraction, and blind source separation (Comon, 1994). Before applying the ICA, we whiten the deep scattering spectrogram by equalizing its covariance matrix eigenvalues, allowing us to disregard patterns' relative amplitudes as much as possible. The number of most relevant features (or independent components) is often unknown and should be inferred, which is explained more in detail in Text S2 in Supporting Information S1.

Lastly, we perform hierarchical clustering in the low‐dimensional feature space built by the independent components. Clustering aims at grouping objects—here defined as data points in a given feature space—based on a similarity or dissimilarity measurement. With a bottom‐up approach of hierarchical clustering, also called agglomerative clustering, all objects start in a singleton cluster and merge to larger clusters until all objects unify in a single cluster (S. C. S. C. Johnson, 1967). A dendrogram depicts this process, representing the inter‐cluster similarity in a cluster‐distance diagram. The similarity measurement, which drives the cluster merging, is often a distance in the feature space between the objects. Thus, the type of distance is the only choice to be made here and determines the structure of the dendrogram. We use Ward's method as a criterion to merge clusters in hierarchical clustering and produce the dendrogram. Clusters are merged with the objective to keep the increase of the total within‐cluster variance minimal (Ward Jr, 1963). This allows to find cluster of various size, which fits the nature of seismic data, where ambient seismic activity often outweighs transient signals. Finally, depending on the truncation distance explored in the dendrogram, one can obtain a different number of clusters. This allows exploring the data set's structure and searching for a cluster of seismic signals related to the ground frost. The dendrogram is unique to hierarchical clustering and the main reason why we choose this clustering algorithm instead of others.

## 4 Cluster of Signals Occurs During Ground Frost

**Figure 3:**
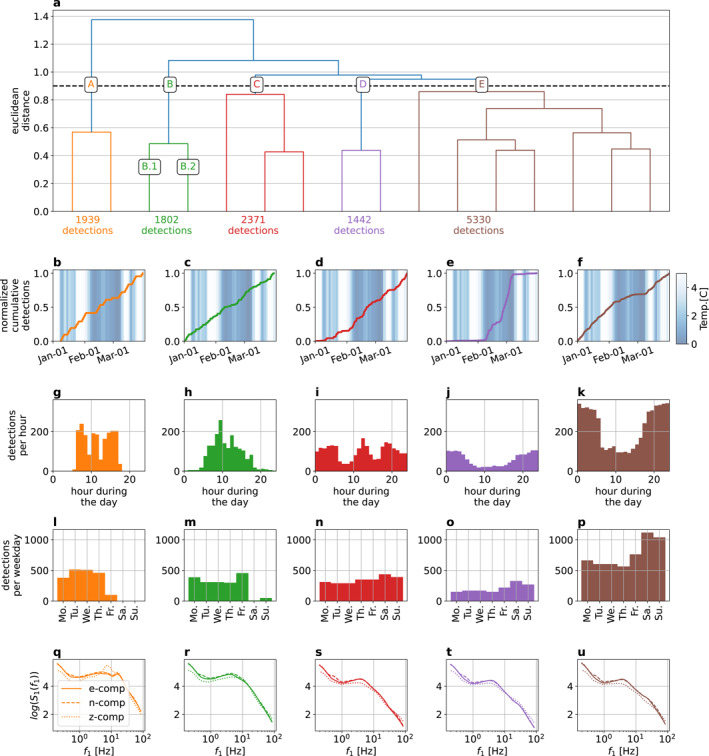
Results of seismic data clustering from the three‐component broadband station WM01 between 1 January 2018 and 1 April 2018. (a) Dendrogram with a truncation distance set to obtain 16 clusters. (b–f) Normalized cumulative detection. (g–k) Daily occurrence. (l–p) Weekly occurrence. (q–u) Averaged first‐order scattering coefficients.

We show a truncated dendrogram of the continuous three‐component seismogram recorded at station WM01 from January to April 2018 in Figure 3a, using a truncation distance to end up with 16 clusters in this case. A data point in the feature space represents 10 min of continuous waveform data without overlap. Moreover, the feature space contains 16 independent components, as a trade‐off between keeping enough information and low dimensionality (see Text S2 and Figure S1 in Supporting Information S1). Note that finding a cluster related to ground frost effects is an exploratory task where we do not know where such a cluster would appear in the dendrogram nor if it even exists. As suggested in Steinmann et al. (2022), we extract a few large clusters at a high distance threshold to overview the whole data set. We can then focus on certain branches in the dendrogram and extract subclusters hierarchically to get a more detailed cluster analysis if needed. In our case, we extract five clusters (hereafter denoted A, B, C, D, and E) at a distance threshold of 0.9 (Figure 3a). In the following lines, we will interpret the clusters and assign meaningful labels with certain inherent clusters properties such as the normalized cumulative detections in time (Figures 3b–3f), the number of detections per hour during the day (Figures 3g–3k), the number of detections per weekday (Figures 3l–3p), and the first‐order scattering coefficients averaged for each input channel (Figures 3q–3u). In particular, the normalized cumulative detections in time can help identify a cluster related to the presence of ground frost since the temperature time series indicate the periods of freezing air temperature. Note that a detection refers to a 10 min window of seismic data which is assigned to one of the five clusters.

Cluster A seems to detect in a linear‐piecewise way, with no relation to the temperature time series or occurrence of ground frost (Figure 3b). This cluster detects only between 05:00 and 18:00 local time from Monday to Friday (Figures 3g and 3i). Note that around 09:00 and 12:00, the detections reach a minimum, coinciding with the typical breakfast and lunch break during workdays. Compared to the other clusters, the averaged first‐order scattering coefficients show larger values for frequencies above 1 Hz with a local maximum around 8 Hz on the vertical component (Figure 3q). The analysis of these parameters indicates that this cluster contains seismic signals related to anthropogenic sources, mainly active during classical labor hours. The gravel pit with trucks in the direct neighborhood of this measuring site could be a possible source (Figure 1a).

Cluster B seems to detect more continuously than cluster A (Figure 3c). It is active during the daytime, with a few detections during the nighttime (Figure 3h). Interestingly, this cluster peaks at 09:00 and 12:00 when cluster A reaches a minimum of detections. The weekdays show clearly more detections than the weekends, with a peak of detection on Fridays when cluster A shows a minimum of detection during the week (Figures 3l and 3m). The averaged first‐order scattering coefficients show similar frequency characteristics as cluster A. However, cluster B indicates no bumps around 8 Hz (Figure 3r). The analysis of cluster B suggests that this cluster also relates to anthropogenic activity. Since it shows elevated activity when cluster A reduces its activity (Fridays and 09:00 and 12:00 local time), it is probably related to a different anthropogenic seismic source. Because cluster B also contains some detections during the nighttime and weekends, it possibly contains seismic signals related to nearby road traffic.

Cluster C is the second‐largest cluster of the whole data set (Figure 3a). It detects irregularly at all hours and all days (Figures 3d, 3i, and 3n). During the morning and afternoon its detection rate decreases (Figure 3i). Moreover, the averaged first‐order scattering coefficients show no particular pattern (Figure 3s). It is unclear what type of seismic signals cluster C contains. We can only note that it is not related to ground frost since its detections rate does not correlate with freezing temperatures.

Cluster D activates mainly during two periods (Figure 3e). At the beginning of February, it accumulates 25% of its size followed by a slight pause. Then, at the end of February and beginning of March it detects the remaining 75% of its total size. The detection periods occur during the coldest temperatures recorded at 5 cm depth. Therefore, cluster D most likely groups seismic signals related to ground frost. Cluster D detects during all hours and all days. However, slightly more detections appear during the weekend and nighttime (Figures 3j and 3o). There are probably two effects that explain this behavior. First, due to colder temperatures, ground frost occurs predominantly at night and so do the associated seismic signals (Figure 1b). Second, due to anthropogenic activity, the seismic wavefield in an urban environment changes significantly between day and night and weekdays and weekends. Thus, the changing wavefield modulates the signature of the ground frost recorded by continuous seismograms. For instance, a seismogram containing seismic signals generated by road traffic during ground frost could be found in cluster B or D. Indeed, inside cluster B, we can identify subcluster B.1 as anthropogenic seismic signals effected by the ground frost (see Figure 3a and Figure S2 in Supporting Information S1). This points out a limitation of clustering: a seismogram containing multiple types of signals is assigned to a single cluster, which oversimplifies the nature of the data and has been already noted by Steinmann et al. (2022). The averaged first‐order scattering coefficients show no clear and distinct pattern (Figure 3t). Cluster D seems different from Cluster A and B due to lower scattering coefficients for higher frequencies. However, it is unclear how cluster D differs from clusters C and E. We can note that the averaged first‐order scattering coefficients do not deliver a unique signature related to these signals.

Cluster E is the largest cluster of the whole data set (Figure 3a). It detects continuously with a decreased detection rate during February when ground frost occurs, with more detections during night and weekends (Figures 3f, 3k, and 3p). Moreover, the cluster shows lower averaged first‐order scattering coefficients at higher frequencies (Figure 3u), distinguishing them from clusters A and B but D. The analysis of cluster E indicates that it groups ambient seismic noise without particular transients and ground frost. In fact, it appears that cluster D and E summarize the stationary ambient wavefield separated only due to the occurrence of ground frost. Indeed, the combined clusters seems to detect almost continuously during weekends and nights (see Figure S2 in Supporting Information S1).

Summarized, the dendrogram delivers a data‐driven overview about the content of the data containing both source and medium effects. We can clearly identify cluster A and B with anthropogenic seismic sources. Inside cluster B we identified a small subcluster containing anthropogenic signals effected by the ground frost. We have reasons to assume that a more detailed cluster solution would reveal a similar subcluster in A. We cannot find a meaningful label for cluster C. The largest part of the data is located within‐cluster E: ambient seismic noise, which is not effected by ground frost. Cluster D seems to be the only cluster related to the freezing of the surface without particular transient signals from anthropogenic activity. The hierarchical clustering approach, together with an interpretation of a cluster solution at a high distance threshold, allowed us to give a detailed analysis of the content of the seismic data. In particular, the cumulative detection curve identifies cluster D as of interest in our study because it relates purely to ground frost. Hence, we do not need to extract a more detailed cluster solution. In the following lines, we analyze how the freezing and thawing process is encoded in the data.

## 5 Disentagling the Ground Frost From the Urban Imprint

**Figure 4:**
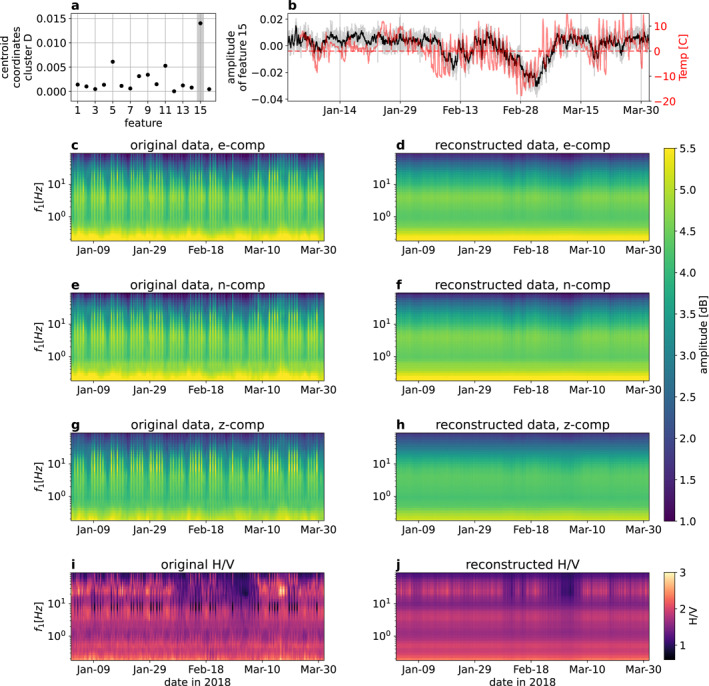
The signature of freezing (a) coordinates of the centroid of cluster D in the eight‐dimensional feature space. (b) Feature 15 as a smoothed time‐series (black) compared to the temperature time‐series recorded above ground (red). The original feature without smoothing is represented in gray. (c, e, and g) Original first‐order scattering coefficients for the east, north and vertical component, respectively. (d, f, and h) Reconstructed first‐order scattering coefficients based solely on feature 15 for the east, north and vertical component, respectively. (i) Ratio between horizontal and vertical components based on the original first‐order scattering coefficients. (j) Ratio between horizontal and vertical components based on the reconstructed first‐order scattering coefficients.

Hierarchical clustering built the dendrogram within the feature space extracted by an ICA from the deep scattering spectrogram (Figure 2). The features likely reveal insights about the signature of cluster D and, thus, about the ground frost signature. Steinmann et al. (2022) already showed that single features retrieved from the scattering coefficients with an ICA could reveal interesting patterns in the seismogram. Therefore, we can likely identify a single feature in our data set that encodes the seismic signature of the ground frost. The geometric center of a cluster in the feature space, also called centroid, can tell us if one feature is more important than other features. In our case, we define the geometric center of a cluster as the mean of its data points in the 16‐dimensional feature space. We note that if all features are equally important in defining a cluster, they should contribute equally to the centroid coordinates. If a few or single features are more important than others, the centroid should have a stronger contribution from them. We calculate the centroid of cluster D and take the modulus, since we are only interested in the amplitude information (Figure 4a). We observe that the centroid of cluster D shows a substantial value for feature 15 (Figure 4a) regarding the other features. This suggests that cluster D is active when large absolute values on feature 15 occur.

We can also observe how feature 15 evolves in time (Figure 4b). Feature 15 shows a significant amplitude decrease at the end of February and the beginning of March. During that time, it seems to mimic the low‐frequent trend of the air temperature with a slight offset in time. The beginning of February and mid‐March show smaller amplitude decreases after a few consecutive nights of freezing air temperature. Unfortunately, we have no ground truth about the occurrence of ground frost. However, we know that the occurrence of ground frost depends on the amount of time and the amplitude of freezing air temperature. Moreover, thawing air temperatures during the day counteract the nightly built‐up of ground frost. A more extended and continuous period of freezing air temperature (like the one at the end of February) results in a thicker layer of ground frost. A colder air temperature can also decrease the temperature inside the layer of ground frost and, thus, increase its stiffness and shear wave velocity (Miao et al., 2019; Zimmerman & King, 1986). These facts, combined with the observation of feature 15 and the air temperature, suggest that this feature tracks the freezing and thawing process of the surface at a high‐resolution timescale of 10 min. We emphasize that feature 15 is an entirely data‐driven product from a three‐component seismogram with minimal processing. In comparison, Steinmann et al. (2021) tracked the same freezing and thawing process with data from two seismic stations, heavier preprocessing, and a time resolution of 2 days.

Since ICA is a linear operator, we can use only feature 15 to reconstruct the scattering coefficients out of the mixing matrix, defined as the pseudo‐inverse of the unmixing matrix (Comon, 1994). This procedure acts as a filter process since we zero all features except feature 15. Due to the large size of first‐ and second‐order scattering coefficients, Figures 4c–4h show only the first‐order original and reconstructed scattering coefficients for all three components. The original coefficients show clearly the urban imprint in the seismic data: fringes appear during daytime and pause at the weekends (Figures 4c, 4e, and 4g). No clear pattern appears during ground frost building periods, such as at the end of February (Figure 4b). The reconstructed coefficients do not contain the fringes due to urban activity since these signals were probably encoded in one of the muted features (Figures 4d, 4f, and 4h). The filtering effect reveals a slight amplitude decrease for the horizontal components at frequencies above 1 Hz during the end of February, coinciding with the coldest period of the data set. During that time, a faint amplitude decrease can also be observed at the vertical component. At times with consecutive cold nights such as at the beginning of February or mid‐March, these decreases are also faintly visible. These observations confirm that the wavefield experiences an energy decrease during ground frost with a discrepancy between horizontal and vertical components. Indeed, the ratio of horizontal and vertical scattering coefficients show a clear broadband high‐frequent decrease at the beginning and end of February for both original and reconstructed data (Figures 4i and 4j). It appears that the broadband decrease in the ratio becomes stronger with increasing time or amplitude of the freezing air temperature. The ratio of horizontal and vertical scattering coefficients resembles the classical horizontal‐to‐vertical‐spectral‐ratio (HVSR) based on the Fourier transform. The question rises if the observed change in the seismic data is due to a changing medium caused by freezing and thawing or due to changes in the seismic sources. First of all, we could argue that a source change would probably effect all three components similarly, which is not our case. Moreover, if a temperature related source would appear, it would probably increase the energy during times of freezing, which also does not fit our observations. In fact, it was shown before that ground frost can cause a broadband decrease in the HVSR for higher frequencies (Guéguen et al., 2017). Our observations suggest that less than 5 cm of ground frost has already an impact on the seismic wavefield. Indeed, models based on the diffusive field assumption (García‐Jerez et al., 2016; Piña‐Flores et al., 2016; Sánchez‐Sesma et al., 2011) confirm an HVSR decrease due to a thin layer of ground frost (see Text S3 and S4, and Figures S3 and S4 in Supporting Information S1). All these arguments suggest strongly that the revealed signature is indeed due to a medium change.

## 6 Conclusion

In this study, we made the first attempts toward inferring blindly medium changes from the wavefield recorded by a single station. For our case study, the medium continuously changes due to surface freezing and thawing, while anthropogenic activity creates a complex and non‐stationary seismic wavefield. An AI‐based approach, based on the deep scattering network, an ICA and hierarchical clustering, helped us explore the seismic data and search for possible patterns induced by the ground frost without assuming how the seismic data could be affected. One of the main outcomes of this study is that the AI‐based approach blindly extracts a feature that isolates the seismic response due to the medium change and mutes other non‐stationary processes. This opens new possibilities to utilize single station data for monitoring purposes, especially in environments with many source and medium processes such as permafrost (e.g., Köhler & Weidle, 2019) or volcanoes. AI‐based strategies could complement other passive seismic methods used for permafrost monitoring (e.g., Cheng et al., 2022; James et al., 2019; Lindner et al., 2021). This could give new insight into the response of permafrost to climate change given the decade‐long availability of single seismic stations near permafrost areas. Future research could also investigate if other types of medium changes (e.g., groundwater fluctuations) could be directly extracted from the seismograms in a data‐driven fashion.

Moreover, the revealed signature combined with the HVSR model indicates that superficial freezing might impact the modal energy distribution. This effect has been observed for other high‐velocity surface layers at engineering sites (O’Neill & Matsuoka, 2005). However, to our knowledge, it has not yet been considered in permafrost studies using passive seismic methods. On the one hand, it could corrupt velocity variation measurements retrieved from surface waves in cross‐correlograms. On the other hand, it would also be an opportunity since more modes increase the amount of information about the subsurface. Future research is needed to understand better the interaction between different surface wave modes in the presence of frozen surface layers.

## Supporting information

Supporting Information S1

## Data Availability

The seismic data were downloaded from Steinmannn et al. (2020) and the temperature data were provided by the Meteorological Institute of Hamburg. The temperature data can be retrieved by contacting the Meteorological Institute of Hamburg through https://wettermast.uni‐hamburg.de/frame.php?doc=Impressum.htm. The main code for calculating the scattering coefficients, features, and linkage matrix can be found under https://zenodo.org/badge/latestdoi/460424596. The work relies heavily on the python packages ObsPy (Beyreuther et al., 2010), scikit‐learn (Pedregosa et al., 2011), and SciPy (Virtanen et al., 2020). The map was produced with map tiles by Stamen Design, under CC BY 3.0. Data by OpenStreetMap, under ODbL.
