# Supplementary material for: AI‐Based Unmixing of Medium and Source Signatures From Seismograms: Ground Freezing Patterns
Source: Geophys Res Lett. 2022 Aug 11;49(15):e2022GL098854. doi: 10.1029/2022GL098854 (PMC9541848; doi:10.1029/2022GL098854)
Supplement: Supplementary file 1 — Supporting Information S1 [file GRL-49-e2022GL098854-s001.pdf]

Supporting Information for "AI-based unmixing of medium and source signatures from seismograms: ground freezing patterns"

René Steinmann[1], Léonard Seydoux[1,2]and Michel Campillo[1]

[1]ISTerre, équipe Ondes et Structures, Université Grenoble-Alpes, UMR CNRS 5375, 1381 Rue de la Piscine, 38610, Gières, France

[2]Department of Earth, Atmospheric and Planetary Sciences, Massachusetts Institute of Technology, Cambridge, MA, USA

Introduction

The seismic data is sampled with 200 Hz. Because the data was retrieved manually from the field, three data gaps of ca. 3 h occur in the dataset. Before applying the hierarchical waveform clustering, the data was demeaned and high-pass filtered with a corner frequency of 0.1 Hz. The data gaps were filled with zeroes. However, the scattering coefficients of the data gaps were removed before the feature selection. The supporting information provides details about:

1. the design of the deep scattering network (Text S1)
2. the number of releveant features retrieved with an ICA (Text S2 and Figure S1)
3. the cumulative detections for subcluster B.1, B.2 and the combination of cluster D and E (Figure S2)

4. the HVSR models with and without a thin layer of ground frost (Text S3 and S4, Table S1, and Figure S3 and S4)

Text S1: Design of deep scattering network

We design a deep scattering network with 36 complex-valued Gabor wavelets in the first layer and 9 Gabor wavelets in the second layer. A modulus operation retrieves real-valued scalograms. The first layer creates 36 scattering coefficients and the second layer creates 324 (as from 36×9) scattering coefficients per sliding window and component. The center frequencies of the first-layer wavelets range from 0.2 to 89 Hz and the center frequencies of the second layer wavelets range from 0.2 to 50 Hz. The number of wavelets was chosen specifically to cover a wide range of frequencies above the oceanic microseism. The upper frequency of the first layer is bounded by the sampling frequency of 200 Hz. The center frequencies are spaced logarithmically with four wavelets per octave in the first layer and one wavelet per octave in the second layer. The sliding window is set to 10 min to mimic the time resolution of the temperature data. In contrast to Steinmann, Seydoux, Beaucé, and Campillo (2022), we apply average pooling instead of maximum pooling to the first and second layer scalograms since we are not searching for transient signals but changes in the ambient seismic wavefield.

Text S2: Extracting the most relevant features

After calculating the deep scattering spectrogram, we apply an ICA to retrieve the most relevant features. The ICA model can be written as:

$$\mathbf{x} = \mathbf{s}\mathbf{A}, \tag{1}$$

where $\mathbf{x} \in \mathbb{R}^{N\times F}$ are the N observations of dimension F, $\mathbf{A} \in \mathbb{R}^{F\times C}$ is the mixing matrix, and $\mathbf{s} \in \mathbb{R}^{C\times N}$ are the C independent components, representing the set of features for hierarchical clustering. Equation 1 considers the observations $\mathbf{x}$ as a linear combination of the independent components $\mathbf{s}$, with the mixing weights gathered in $\mathbf{A}$. In our case, $\mathbf{x}$ are the whitened scattering coefficients. Setting the number of independent components is an exploratory task that can be seen as a trade-off between keeping the dimensionality low for clustering and retaining the most crucial data information. We use the reconstruction loss $\epsilon(C)$ between the original data $\mathbf{x}$ and the reconstructed data $\hat{\mathbf{x}}^{(C)}$, based on the C independent components, as a guideline for choosing an optimal number for C. The reconstruction loss is defined as following:

$$\epsilon(C) = \frac{\sum_{i=0}^{N} |x_i - \hat{x}_i^{(C)}|}{N}. \tag{2}$$

Figure S1 depicts the reconstruction loss $\epsilon(C)$ for an increasing number of independent components C. The reconstruction loss decreases rapidly with the first 14 components. With more than 14 components, the rate of error decrease becomes smaller and almost linear. However, a small jump occurs from 14 to 16 components. Therefore, 16 independent components, marking a kink in the reconstruction error curve, seem like a good choice to us and are the basis for building the linkage matrix for the dendrogram.

Text S3: Inverting for a 1D velocity model

To forward model the effect of ground frost on the HVSR, we need a 1D velocity model with the shear wave velocity v_s, the compressional wave velocity v_p, the thickness of the layer h and the density ρ. Steinmann, Hadziioannou, and Larose (2021) provides a 1D

velocity model to a depth of less than 30 m based on a shear wave refraction profile. The forward modelled HVSR based on this velocity model together with the observed HVSR at the three stations at 15 April 2018 are shown in Figure S3. We chose this day for an HVSR measurement for two reasons. Firstly, the time of the year and the temperature data suggest that we do not have any ground frost (Figure 1a). Secondly, it is a Sunday and, thus, we have better conditions for an equipartitionned wavefield without anthropogenic activity (Figure 3). It is clear that the modelled HVSR does not fit the observations. Since the two resonance peaks below 1 Hz do not occur in the modelled HVSR, it appears that the velocity model is not deep enough. To update the velocity model, we invert the HVSR measurements based on the diffusive field assumption (Piña-Flores et al., 2016). We invert for a three-layer model with the observed HVSR between 0.1 and 1 Hz to fit the two resonance peaks. The higher frequency content seems unreliable, since the variations between the stations are too large given the fact that they are only 100 m apart (see map in Figure 1a). These variations at higher frequencies can be the result of different installation types. WM01 and WM02 are placed on a concrete slab while WM03 is inside a shed. We constrain the range of possible shear wave velocity of the first layer with the values given in Steinmann et al. (2021). The updated and deeper velocity model fits better the observations and, thus, is utilized for modelling the effect of the ground frost. The values of the updated model are presented in Table S1.

Text S4: Modelling the effect of a frozen surface on the HVSR

We model the effect of ground frost on the HVSR based on a 1D velocity model and diffuse wavefield assumption (Sánchez-Sesma et al., 2011; García-Jerez et al., 2016). Firstly,

we derive a 1D velocity model from the inversion of H/V measurements (Piña-Flores et al., 2016) and constraints from a shear wave refraction profile (Steinmann et al., 2021). To evaluate the effect of ground frost, we insert a centimeter thick high-velocity layer at the surface of the 1D model. Different thicknesses and shear wave velocities account for different scenarios of the ground frost. The shear wave velocity of the ground frost depends strongly on the temperature and composition of the soil. A silt-clay mixture with a high water content as in our case can reach the eight-fold of its shear wave velocity with temperatures below $-8\,^{\circ}\mathrm{C}$ (Miao et al., 2019). Through the shear wave velocity and a constant Poisson's ratio of 0.33 (Zimmerman & King, 1986), we define the compressional wave velocity. We neglect changes in the density and set it to $2000\,\mathrm{kg\,m^{-3}}$ for all layers.

Figure S4 shows the HVSR for different scenarios of ground frost and different number of considered surface waves modes. All models confirm the qualitative observation that the HVSR experiences a broadband decrease above 1 Hz due to a layer of ground frost with a certain thickness and increased shear wave velocity. Apart from the broadband decrease at higher frequencies, the two resonance peaks below 1 Hz do not seem to be effected. With increasing thickness and shear wave velocity the decrease is more pronounced and the maximum decrease moves to lower frequencies. Note that both parameters show a similar effect on the HVSR. Thus, it is difficult to disentangle the two effects in actual observations. We observe this scenario at the end of February and beginning of March marking the coldest and also the longest period of freezing air temperature (Figure 1b). During that time, the horizontal component and the HVSR experience the strongest decrease. However, we cannot say if an increasing thickness or decreasing temperature

dominates the process. The number of surface modes considered in the wavefield has also an effect on the pattern of decrease. It has already been shown that large stiffness contrasts or reversal of velocity layers – that is high-velocity layer over low-velocity layer – can cause modal energy pertubation and dominant higher modes (O'Neill & Matsuoka, 2005). Freezing the soil from the surface downwards causes a reversal of velocity layers and might lead to modal energy pertubation. The broadband high-frequent HVSR decrease and its dependence on the number of modes suggest that this effect occurs. This would be important to consider when passive image interferometry is used for monitoring permafrosts. Dominant higher modes could appear on cross-correlograms during times of refreezing in autumn and corrupt measurements of velocity variations. A proper wavefield analysis would be needed to understand this process better, however, it is out of the scope of this work and, thus, subject to future research.

Overall, the model brings interesting insights to our observations retrieved from the seismic data. The observations and model agree qualitatively on a broadband high-frequent HVSR decrease due to ground frost. The decrease is more pronounced for deeper and colder ground frost. Moreover, the model shows that it is difficult to entangle the interaction between the thickness and temperature of the ground frost and surface wave modes present in the wavefield. It is also clear that the HVSR of the seismic data contains many different source and medium effects (Figure 4i) and, thus, the diffusive wavefield assumption is not valid for the data. This highlights the strength of our data-driven approach, which isolates a pattern in the continuous seismograms related to the freezing and thawing process despite all the other source and medium effects affecting the data.

References

García-Jerez, A., Piña-Flores, J., Sánchez-Sesma, F. J., Luzón, F., & Perton, M. (2016). A computer code for forward calculation and inversion of the h/v spectral ratio under the diffuse field assumption. *Computers & geosciences*, *97*, 67–78.

Miao, Y., Shi, Y., Zhuang, H., Wang, S., Liu, H., & Yu, X. (2019). Influence of seasonal frozen soil on near-surface shear wave velocity in eastern Hokkaido, Japan. *Geophysical Research Letters*, *46*(16), 9497 – 9508.

O'Neill, A., & Matsuoka, T. (2005). Dominant higher surface-wave modes and possible inversion pitfalls. *Journal of Environmental & Engineering Geophysics*, *10*(2), 185–201.

Piña-Flores, J., Perton, M., García-Jerez, A., Carmona, E., Luzón, F., Molina-Villegas, J. C., & Sánchez-Sesma, F. J. (2016). The inversion of spectral ratio h/v in a layered system using the diffuse field assumption (dfa). *Geophysical Journal International*, ggw416.

Sánchez-Sesma, F. J., Rodríguez, M., Iturrarán-Viveros, U., Luzón, F., Campillo, M., Margerin, L., ... Rodriguez-Castellanos, A. (2011). A theory for microtremor h/v spectral ratio: application for a layered medium. *Geophysical Journal International*, *186*(1), 221–225.

Steinmann, R., Hadziioannou, C., & Larose, E. (2021). Effect of centimetric freezing of the near subsurface on rayleigh and love wave velocity in ambient seismic noise correlations. *Geophysical Journal International*, *224*(1), 626–636.

Steinmann, R., Seydoux, L., Beaucé, E., & Campillo, M. (2022). Hierarchical exploration

of continuous seismograms with unsupervised learning. *Journal of Geophysical Research: Solid Earth*, *127*(1), e2021JB022455.

Zimmerman, R. W., & King, M. S. (1986). The effect of the extent of freezing on seismic velocities in unconsolidated permafrost. *Geophysics*, *51*(6), 1285–1290.

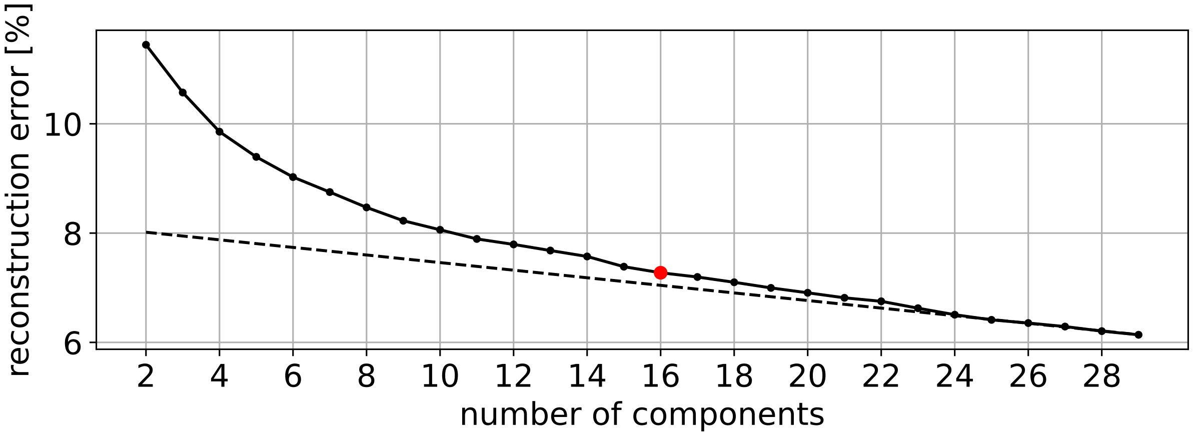

Figure S1. Reconstruction error for ICA-models with different number of independent components. The red dot marks the model we choose for further analysis. The dashed line fits a linear function based on the last seven points.

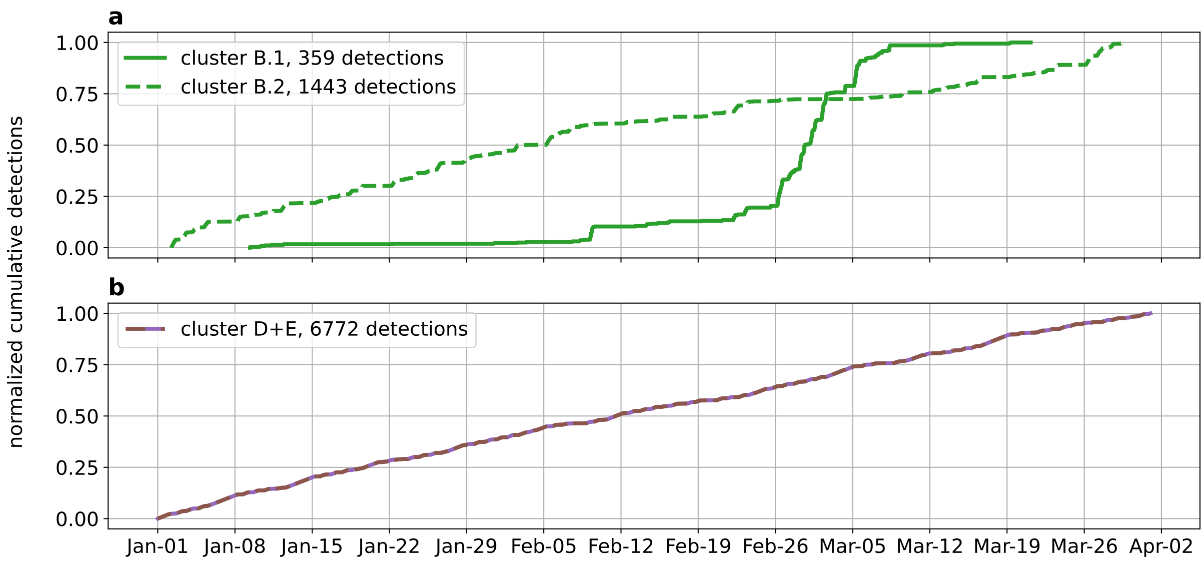

Figure S2. Normalized cumulative detections for other cluster solutions. Normalized cumulative detections for subcluster B.1 and B.2 **(a)** and the cluster-combination of D and E **(b)**. Note that each tick at the x-axis marks a Monday.

h [m]	v_s [m/s]	v_p [m/s]	ρ [g/cm^3]
172.82	394.54	1255.93	2000
611.60	520.96	2075.66	2000
∞	947.09	4250.25	2000

Table S1. 1D model of the subsurface at the measuring site based on the inversion of the HVSR with the diffusive field assumption

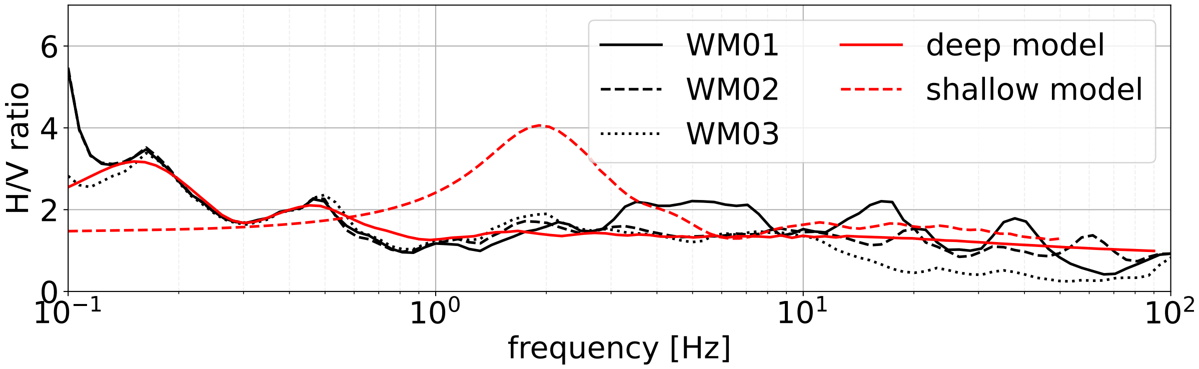

Figure S3. The observed HVSR at all three stations, the modelled HVSR based on the velocity model given in Steinmann et al. (2021) as the dashed red line and the modelled HVSR based on the inversion of the HVSR as the red solid line.

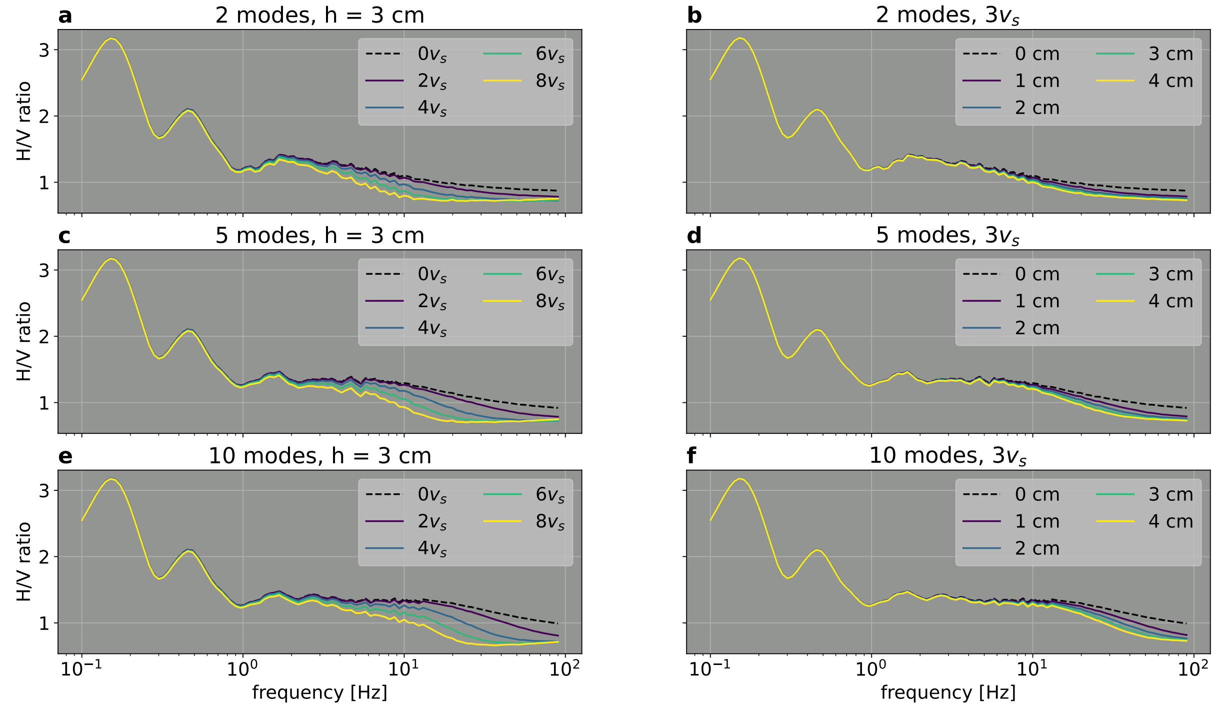

Figure S4. (**a,c,e**) The HVSR in the presence of a 3 cm thick frozen surface layer with varying shear wave velocities and varying number of Rayleigh and Love wave modes. The shear wave velocity of the frozen layer ranges between two-fold and eight-fold of the shear wave velocity of the first layer in the 1D model. The model without a frozen layer is depicted as a black dashed line. (**b,d,f**) The HVSR in the presence of a frozen surface layer with a thickness ranging from 1 to 4 cm and varying number of Rayleigh and Love wave modes. The shear wave velocity is fixed to the three-fold shear wave velocity of the first layer. The model without a frozen layer is depicted as a black dashed line.
